# Characteristics and population estimates of unpaid end of life carers: An observational study

**DOI:** 10.1177/02692163251366090

**Published:** 2025-09-07

**Authors:** Clare Gardiner, Arthur Juet, Edward JD Webb, Juliet Stone

**Affiliations:** 1 School of Allied Health Professions, Nursing & Midwifery, The University of Sheffield, Sheffield, UK; 2 Academic Unit of Health Economics, Leeds Institute of Health Sciences, University of Leeds, Leeds, UK; 3 Centre for Research in Social Policy, Loughborough University, Loughborough, UK

**Keywords:** Unpaid carers, family carers, end of life, palliative care, poverty

## Abstract

**Background:**

Improving support for unpaid carers is a policy priority internationally, yet there are few reliable population estimates on numbers of end of life carers, and little is known about the demographic characteristics of this group.

**Aim:**

(1) Estimate the number of unpaid end of life carers in the UK; (2) Describe demographic characteristics of this group.

**Design:**

An observational study using data from the UK Household Longitudinal Survey (Understanding Society), Health Survey for England and the Office for National Statistics to estimate the number of end of life carers in the UK. Understanding Society was used to explore characteristics of end of life carers including poverty before and after bereavement.

**Participants:**

Understanding Society collects annual data on around 40,000 households in the UK, including carers.

**Results:**

Data from Understanding Society suggests there are 150,000–180,000 unpaid end of life care in the UK each year, while data from Health Survey for England suggests a higher estimate at 570,000–775,000 carers. End of life carers are more likely to be older and female. There is an increase in the percentage of carers falling into poverty one year after they provided care.

**Conclusions:**

These analyses have provided the first estimate of the number of end of life carers in the UK, using methods which are replicable in other countries. Our data provide a useful benchmark both for the UK and for other comparable high income countries. Many end of life carers are living in poverty, including after bereavement, showing a need for policy initiatives to provide support.

**What is already known about the topic?**

- Caring for someone at the end of life has significant negative impacts for the carer including worse psychological and physical health, financial burden and employment impacts.
- There is limited evidence on population estimates and characteristics of end of life carers internationally.
- Improving support for unpaid carers in a policy priority internationally, yet data to inform policy developments are lacking.

**What this paper adds?**

- Using Understanding Society data we estimate that a minimum of 150,000–180,000 people in the UK each year experience caring for someone within their household at the end of life. Data from Health Survey for England suggests that between 570,000 and 775,000 people in the UK provide care for someone at the end of life.
- The higher estimate indicates that over 1% of the UK population are end of life carers, providing a useful benchmark both for the UK and for other comparable high income countries.
- End of life carers are more likely to be older and more likely to be female than other household carers.
- There is a notable rise in poverty in the first year of bereavement.

**Implications for practice, theory or policy**

- Substantial numbers of individuals in the UK are providing unpaid end of life care each year.
- Whilst the data we present are from the UK, the methods we use are transferable to other countries with availability of similar population data.
- Policy initiatives are required to provide improved support for end of life carers, particularly financial support.

## Introduction

Unpaid carers play a vital role in supporting people towards the end of life. They are often family members or partners, but may also include friends, neighbours and other individuals providing unpaid support to someone approaching the end of life. Evidence shows that patients who are supported by an unpaid carer are more likely to have better outcomes at the end of life, and have lower costs linked to health care service use.^1–3^ However, caring for someone at the end of life has significant negative impacts for the carer, including worse psychological and physical health,^4^ financial burden and employment impacts.^5^

International estimates vary but most high income countries report unpaid carer prevalence at between 9% and 15% of the population.^6–8^ In low and middle income countries, limited availability of formal healthcare services means carer prevalence is expected to be even higher.^9^ Accurate population estimates of unpaid carers are difficult to establish, in part due to the proliferation of definitions for the term ‘carer’, but also because many people who provide care do not self-identify as such in population surveys and censuses. This leads to substantial variation in estimates, which impacts on the provision of support for unpaid carers.

Evidence on population estimates and characteristics of unpaid end of life carers is particularly sparse, yet these data are crucial for informing the delivery of palliative and end of life care services, which rely heavily on the support provided by unpaid carers. In the UK, one study suggests that between 5% and 7% of the population may be providing unpaid care to someone at the end of life, however evidence is limited by small sample sizes and unrepresentative data sources.^10,11^ International estimates on the number of end of life carers are similarly scarce, and although the vital role of carers is well established, there is limited evidence on the characteristics of end of life carer populations across the world, particularly in low and middle income countries.

Over recent years a growing number of international studies have confirmed the financial burden and financial support needs associated with unpaid caregiving at the end of life.^5,11–15^ Recent societal and economic shocks – including the COVID-19 pandemic and the cost of living crisis across the Global North – have made this situation even more challenging for carers and their families.^16^ This is reflected in poverty statistics. In the UK, the poverty rate has been consistently higher among people who provide unpaid care than for those who are not carers, and this gap has been widening.^17,18^ There has been a particularly sharp rise in recent years; in 2021/2022 around 28% of carers were estimated to be living below the poverty line, compared with 20% of non-carers.^17^

Internationally, research has confirmed that unpaid end of life carers are not receiving adequate financial support.^19,20^ A prerequisite for developing effective interventions and support for end of life carers is information about their specific needs and characteristics. However, we lack reliable population level estimates on the numbers of unpaid end of life carers, and little is known about the demographic characteristics of this group or how they may differ from the rest of the carer population.

## Methods

The aims of this study were to: (1) establish a population level estimate of the number of unpaid end of life carers across the UK; (2) describe key demographic characteristics of this group.

### Data

#### United Kingdom (UK) Household Longitudinal Survey (Understanding Society)

The UK Household Longitudinal Survey, also known as Understanding Society, is a longitudinal survey of a representative sample of around 40,000 UK households.^21^ It collects information in annual waves (starting in 2009) on a range of topics including employment, finances and mortality. To identify unpaid end of life carers we selected Understanding Society participants who met the following criteria: (i) aged >16 years; (ii) identify as an unpaid carer for someone aged >16 years living in the same household; (iii) where the person being cared for dies prior to the next survey wave. Understanding Society waves from 2009 to 2020 were included.

#### Office for National Statistics

Population data were obtained from the Office for National Statistics UK census data. The census is conducted every 10 years in the UK, most recently 2021.^22^

#### Health Survey for England

Health Survey for England is an annual repeated cross-sectional survey of a representative sample of the English population. Around 8000 adults take part in the survey each year.^23^ In 2013 and 2017, participants were asked if anyone close to them had died of a terminal illness in the past 5 years, and the year of that persons’ death (2013 only). Participants were asked how often they provided care, with response options of daily, occasional/intermittent and rarely in 2013, and options of every day, at least once a week, at least once a month and less than once a month in 2017. End of life carers were defined as those who reported providing personal care to a person who had died in the previous 5 years. ‘Frequent’ end of life carers were defined as participants who provided daily or occasional/intermittent care (in 2013), or who provided care every day or at least once a week (in 2017).

### Analysis

All analyses were conducted in 2024. Using Understanding Society data we identified the proportion of participants who provided end of life care to someone within their household. This was combined with data from the Office for National Statistics on the UK population size to create estimates of the number of end of life carers annually. These estimates were considered to represent the *minimum* numbers of end of life carers in the UK. Due to the nature of the Understanding Society data, only unpaid end of life carers who care for someone within their household could be identified. Many more individuals will provide end of life care to people outside of their own household. In addition, Understanding Society is not able to capture all short episodes of end of life care that is, where a participant takes on a caring role after one wave of data collection, but their dependent dies before the next wave of data collection. To address these limitations, we also used comparative data from two editions of the Health Survey for England to create separate estimates of the number of end of life carers. Health Survey for England has the advantage that it captures end of life caring outside the household, as well as short episodes of care. However, it also has disadvantages including not covering the whole of the UK, having fewer participants than Understanding Society and providing only cross-sectional data from two years, whereas Understanding Society allows tracking of participants between 2009 and 2020. In addition, Health Survey for England asks respondents about end of life caring retrospectively, and provides less detailed information about participants’ financial situation.

#### UK Household Longitudinal Survey

To estimate the number of end of life carers in the UK, we calculated the proportion of Understanding Society respondents who were end of life carers in each year, with sample weights used to ensure that estimates were representative of the UK population. We then multiplied the proportion of end of life carers by the size of the UK population in that year, as estimated by the Office for National Statistics. Understanding Society data prior to 2012 was discarded as initial analysis revealed that the number of deaths observed in the first three years were significantly lower than the UK mortality rate. This would imply underestimating the number of deaths and therefore also of end of life carers.

We summarised characteristics of end of life carers and compared them with the general population and with carers for people within the same household but not at the end of life (termed ‘household carers’). This analysis used all data from 2009 to 2020 since the characteristics of end of life carers were representative of the population throughout this period.

Finally, we studied the fraction of end of life carers in poverty before, during and after the year in which they experienced being an end of life carer. We used two measures of poverty. First, the UK government measure of households in relative low income,^24^ which is those below 60% of the median equivalised household income. Second, the Social Metric Commission poverty index which includes ‘inescapable costs’, such as childcare and additional costs of disability.^25,26^

#### Health Survey for England

Estimates of the number of end of life carers were created by calculating the fraction of end of life carers among Health Survey for England respondents, multiplying by the population size, then dividing by 5 to provide annualised estimates. Estimates were created based on all end of life carers and those who provided end of life care on a ‘frequent’ basis. To mitigate recall bias, additional estimates were provided based only on Health Survey for England 2013 respondents who indicated the care dependent died in the previous calendar year that is, 2012. Summary statistics about end of life carers were also calculated.

## Results

### Understanding society

**Figure 1.**
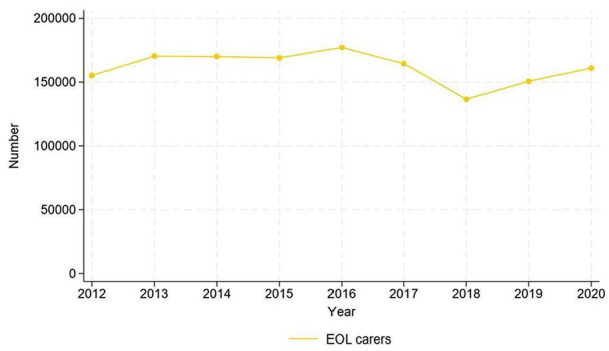
Number of end of life carers in the UK in the years 2012–2020 based on Understanding Society data.

The estimated number of end of life carers across the UK, based on Understanding Society data, varied between 150,000 and 180,000 per year. Figure 1 shows the estimated number of end of life carers over time in the UK.

**Figure 2.**
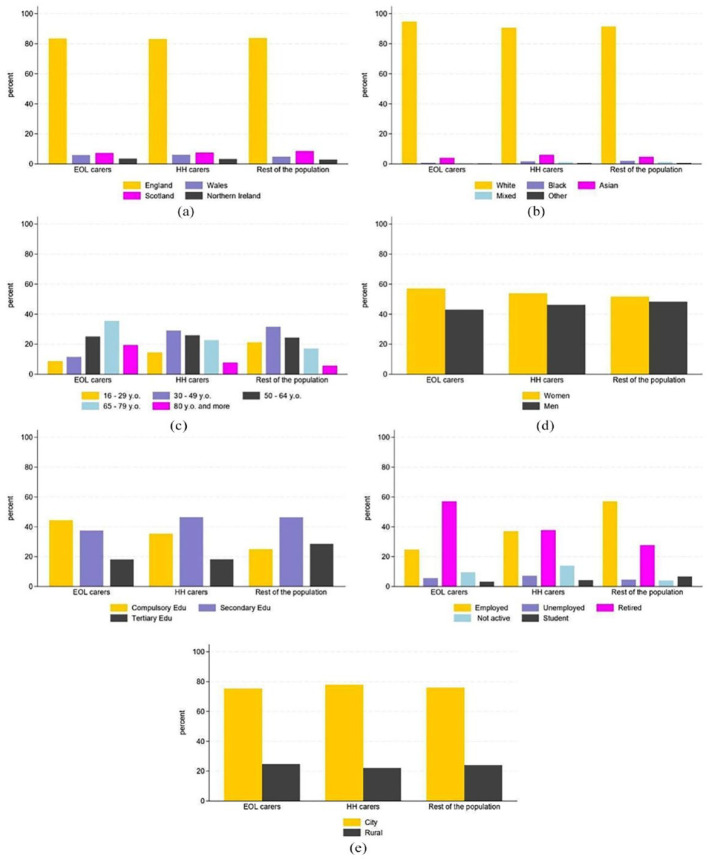
Comparison of socio-demographic distribution between end of life carers, household carers and the rest of the population: (a) comparison of country distribution, (b) comparison of ethnicity distribution, (c) comparison of age distribution, (d) comparison of gender distribution, (e) comparison of education level distribution, (f) comparison of employment status distribution and (g) comparison of urban status distribution.

Figure 2 illustrates the characteristics of end of life carers, household carers and the rest of the UK population. No significant differences were found between groups for country of residency, ethnicity or living in urban vs rural areas. However, end of life carers were significantly older than both the general population (79% vs 48% aged >50 years) and household carers (79% vs 57% aged >50 years). There were significantly more females in the end of life carer group (57% vs 52% of the general population and 54% of household carers). Individuals with lower education levels are also over-represented among end of life carers compared to other groups (44% left school at the minimum age compared with 26% of the general population and 35% of household carers). This difference may be due to end of life carers being older. Figure 2(f) shows a higher frequency of retired individuals among end of life carers, as might be expected with an older population. (See Tables A and B in Supplemental Materials for full details and for statistical differences.)

**Figure 3.**
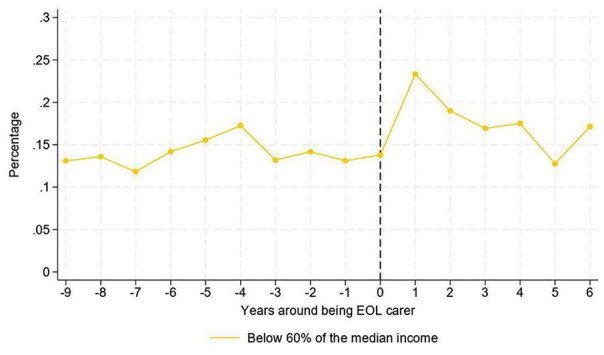
Percentage of individuals below 60% of the median income around the end of life carer event.

**Figure 4.**
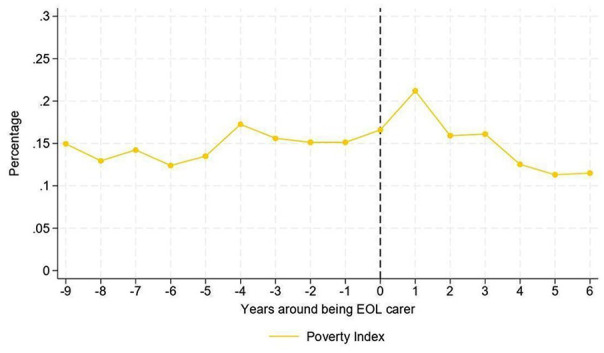
Percentage of individuals above the Social Metric Commission poverty threshold around the end of life carer event.

The number of end of life carers in poverty ranged between 20,000 and 40,000 depending on year and measure of poverty used. Figures 3 and 4 show the percentage of end of life carers in poverty in the years before and after providing end of life care. The figures show an increase in the percentage of individuals falling into poverty one year after they provided end of life care, compared to the wave in which people experienced end of life caring, with increases of 9 percentage points (pp) in households earning <60% of median income (Figure 3) and 5 pp in poverty according to the Social Metric Commission index (Figure 4). However, we observe a rapid return to the levels prior to the event, suggesting that if there is a delayed effect of being an end of life carer observed during the following survey wave, this effect would be temporary.

### Health Survey for England

In the Health Survey for England, 666 (6.1%) of 10,980 participants in 2013 and 524 (5.2%) of 9982 in 2017 had been end of life carers in the last five years, with 588 (5.3%) and 446 (4.5%) respectively doing so on a frequent basis.

Estimates of the number of end of life carers in the UK are given in Table 1. Numbers range from 476,000 to 651,000, with estimates higher in 2013 compared to 2017, and lower when based only on people who provided frequent personal care. Estimates based only on people who reported providing care to someone who died in the previous calendar year were higher than if based on the annual average of those who provided care to someone who died during the previous five years. Extrapolating the Health Survey for England estimates to the whole of the UK, England represents around 84% of the population of the UK and we assume that the same proportion of people provide end of life care in other parts of the UK as in England.^22^ Using annualised data from (as this is available for both 2013 and 2017) this would imply there are between 686,000 and 762,000 end of life carers in the whole of the UK.

**Table 1.** Estimated number of end of life carers in the UK in 2013 and 2017.

Estimate based on respondents who	Health survey for England 2013		Health survey for England 2017	
	*N*	95% CI	*N*	95% CI
Provided any end of life care in previous five years (annualised)	640,000	(590,000–690,000)	576,000	(524,000–629,000)
Provided frequent end of life care in previous five years (annualised)	553,000	(507,000–599,000)	476,000	(429,000–523,000)
Provided any end of life care in previous calendar year	651,000	(539,000–771,000)		
Provided frequent end of life care in previous calendar year	573,000	(471,000–682,000)		

CI: confidence interval.

Confidence intervals calculated using bootstrapping with 10,000 iterations.

## Discussion

### Main findings/results of the study

To the best of our knowledge, this research has produced the first population level estimates of the number of unpaid end of life carers in the UK. The estimate from Understanding Society suggests that a minimum of 150,000–180,000 people in the UK each year experience caring for someone within their household at the end of life. The estimate from Health Survey for England suggests that between 570,000 and 775,000 people in the UK provide care for someone at the end of life. The higher estimate from Health Survey for England suggests that just over 1% of the UK population are end of life carers, providing a useful benchmark both for the UK and for other comparable high income countries. End of life carers are more likely to be older, female and have lower education levels, and there is an increase in the percentage of carers falling into poverty after providing end of life care.

### What the study adds?

Whilst the data we present are from the UK, the methods we use are transferable to other countries with similar population surveys, for example the Survey of Health, Ageing and Retirement in Europe (SHARE), the Australian National Health Survey or the Canadian Community Health Survey.^27–29^ However, some requirements of the data should be noted, as not all surveys will be suitable for exploring characteristics of end of life carers. The main challenge with establishing estimates of end of life carers internationally is reliably identifying the end of life carer population, and this usually requires either longitudinal data (ie Understanding Society) or data reliant on retrospective report (ie Health Survey for England). In addition, surveys need to collect data on carer status (ie asking respondents if they have care responsibilities). Some data on the care dependent is also necessary to confirm that: (i) the care dependent died and; (ii) they received unpaid care prior to death. If these requirements are met then our methods can be adopted to establish estimates of end of life carer populations worldwide. However, the difference between our two estimates from Understanding Society and Health Survey for England emphasises the complexity of capturing reliable data on end of life caring status, and highlights a need for research to further develop methods which can better account for the challenges we describe above. Capturing and recording carer data may be particularly challenging in low and middle income countries, which may lack the necessary infrastructure to record and store necessary data. Innovative use of technology may be one option for improving data capture in this context, and has been used successfully to support health care delivery and data capture.^30^

The research has given insight into the characteristics of the end of life carer community, and in what ways they resemble, and differ from, other household carers. In some ways end of life carers resemble the general population for example, in ethnicity and urbanity. On the other hand, there was a significant overrepresentation of females in the end of life carer group compared with other carers, a concerning finding given evidence highlighting a range of gender inequalities in end of life care.^31^ In addition, end of life carers were found to be older on average than carers in general, who in turn were older than the general population.

In terms of end of life carers’ financial outcomes, a large number were found to be living in poverty, a finding which was robust to the measure of poverty used. This supports previous research highlighting financial burden among end of life carers^5^ and echoes findings from a recent UK report which found over 90,000 people at the end of life were experiencing poverty.^23^ The analysis also shows an increase in the fraction of end of life carers in poverty in the year following their experience of end of life caring. While this study does not seek to establish a causal link between end of life caring and poverty, it provides an early indication that carers require continued support into bereavement and beyond. This finding requires further investigation to explore the causes of poverty after bereavement which may include funeral costs, loss of welfare/benefit payments and ongoing impacts on carer employment. This finding supports previous research that has suggested financial support for carers internationally is inadequate, with urgent reforms needed.^6,32^

The analysis of carers’ financial outcomes requires much further investigation. For example, our findings suggest end of life carers are older than the general population, meaning they are less likely to have financial commitments such as mortgage payments or dependent children. Further research should carefully control for age and other characteristics and should consider analysis strategies that will reveal the causal effect of being an end of life carer.

We are confident that this study has established the minimum number of end of life carers in the UK, focussing on those providing care within the household. However, this study has also shown that it is very challenging to establish a rigorous estimate of the total number of end of life carers with currently available data sources. We have highlighted several issues that should be carefully considered in future research, both in the UK and internationally.

### Strengths and weaknesses/limitations of the study

Whilst Understanding Society is a comprehensive and representative dataset, certain limitations should be acknowledged. Our approach only includes carers who cohabit with their care dependent. As Understanding Society data is only collected annually, it may not capture all ‘short episodes’ of care. Understanding Society and Health Survey for England rely on voluntary responses from participants and therefore it is possible that not all deaths are captured. Understanding Society does not capture data on cause of death so we are unable to distinguish care dependents who died from their underlying condition from those who died from unrelated causes (e.g. accidental deaths). For this reason we use the term ‘end of life’ carers rather than carer of someone with ‘terminal illness’.

We address these limitations in part by complementing our findings based on Understanding Society data with estimates from another data source, Health Survey for England, which includes end of life carers for people outside their household and also captures short episodes of caring. However, Health Survey for England also has limitations, such as not covering the whole of the UK, a smaller sample size and possibility of recall bias. Finally, our study is limited by only focussing on the UK, and generalisability to other countries may be limited.

## Conclusion

This initial analysis has provided the first estimate of the number of end of life carers in the UK, with a minimum of at least 150,000–180,000 people providing care to someone within their household at the end of life each year. The total number of end of life carers is likely to be much higher, and could be as high as 775,000. This suggests that over 1% of the UK population may be providing end of life care. Our methods could be adapted for other countries which routinely collect health survey data, providing a mechanism for identifying and characterising end of life carer populations worldwide. Many end of life carers are living in poverty, and there is a notable rise in poverty in the first year of bereavement, showing a need for policy initiatives to provide support. Future research should further investigate the financial impact of end of life caring, in order to identify where support is most needed and to inform policy.

## Supplemental Material

sj-docx-1-pmj-10.1177_02692163251366090 – Supplemental material for Characteristics and population estimates of unpaid end of life carers: An observational study Supplemental material, sj-docx-1-pmj-10.1177_02692163251366090 for Characteristics and population estimates of unpaid end of life carers: An observational study by Clare Gardiner, Arthur Juet, Edward JD Webb and Juliet Stone in Palliative Medicine
