# Supplementary material for: Characteristics and population estimates of unpaid end of life carers: An observational study
Source: Palliat Med. 2025 Sep 7;39(10):1091–8. doi: 10.1177/02692163251366090 (PMC12640358; doi:10.1177/02692163251366090)

Table A: Comparison of EOL carers with the rest of the population ( excluding household carers)


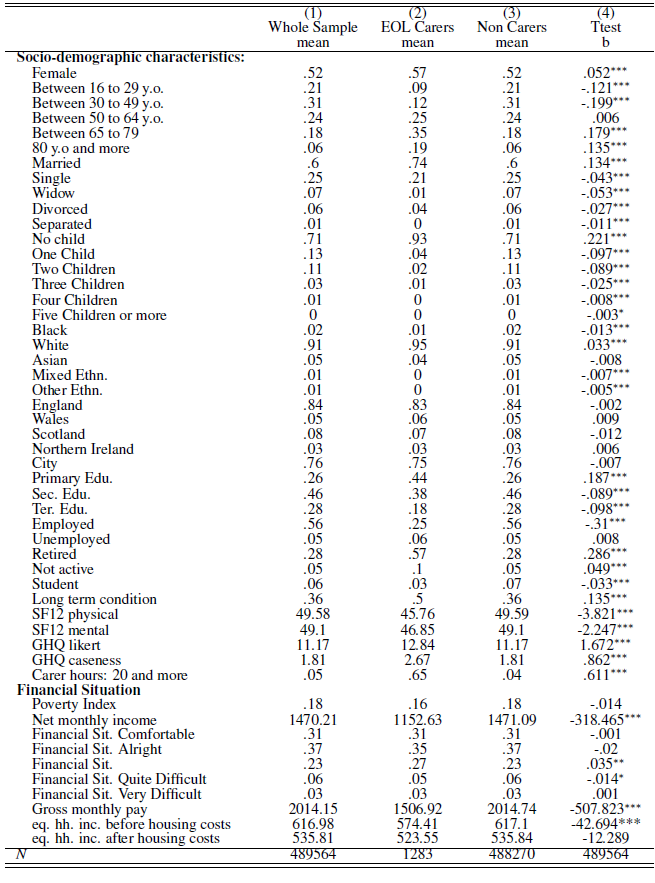


Table B: Comparison of EOL carers with household carers


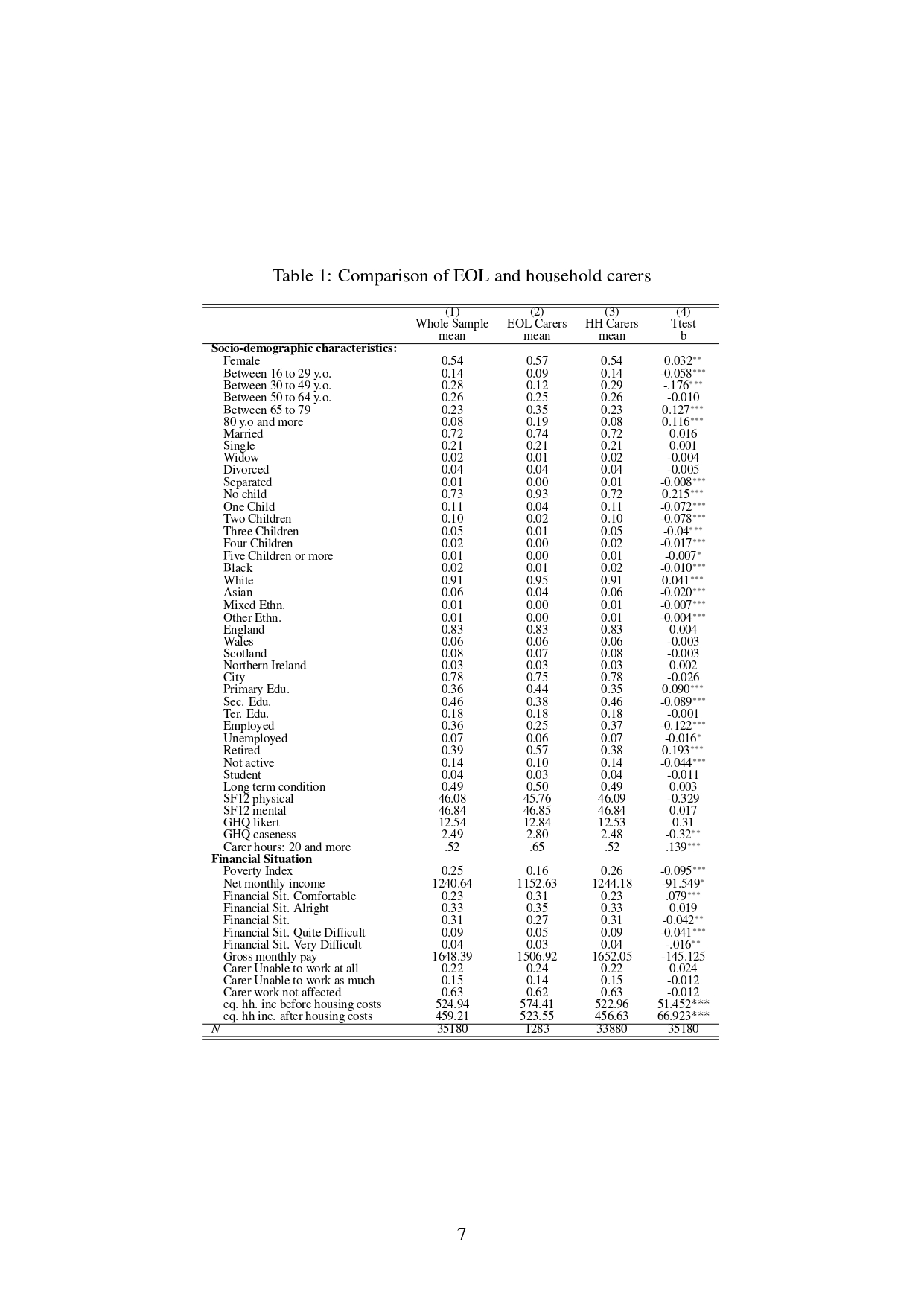

Supplement: sj-docx-1-pmj-10.1177_02692163251366090 – Supplemental material for Characteristics and population estimates of unpaid end of life carers: An observational study [file sj-docx-1-pmj-10.1177_02692163251366090.docx]
